# TGF-Beta Suppresses VEGFA-Mediated Angiogenesis in Colon Cancer Metastasis

**DOI:** 10.1371/journal.pone.0059918

**Published:** 2013-03-25

**Authors:** Liying Geng, Anathbandhu Chaudhuri, Geoffrey Talmon, James L. Wisecarver, Jing Wang

**Affiliations:** 1 Eppley Institute for Research in Cancer and Allied Diseases, University of Nebraska Medical Center, Omaha, Nebraska, United States of America; 2 Department of Pathology and Microbiology, University of Nebraska Medical Center, Omaha, Nebraska, United States of America; University of Texas Health Science Center, United States of America

## Abstract

The FET cell line, derived from an early stage colon carcinoma, is non-tumorigenic in athymic nude mice. Engineered FET cells that express TGF-α (FETα) display constitutively active EGFR/ErbB signaling. These cells readily formed xenograft tumors in athymic nude mice. Importantly, FETα cells retained their response to TGF-beta-mediated growth inhibition, and, like the parental FET cells, expression of a dominant negative TGF-beta type II receptor (DNRII) in FETα cells (FETα/DNRII) abrogated responsiveness to TGF-beta-induced growth inhibition and apoptosis under stress conditions *in vitro* and increased metastatic potential in an orthotopic model *in vivo*, which indicates metastasis suppressor activity of TGF-beta signaling in this model. Cancer angiogenesis is widely regarded as a key attribute for tumor formation and progression. Here we show that TGF-beta signaling inhibits expression of vascular endothelial growth factor A (VEGFA) and that loss of autocrine TGF-beta in FETα/DNRII cells resulted in increased expression of VEGFA. Regulation of VEGFA expression by TGF-beta is not at the transcriptional level but at the post-transcriptional level. Our results indicate that TGF-beta decreases VEGFA protein stability through ubiquitination and degradation in a PKA- and Smad3-dependent and Smad2-independent pathway. Immunohistochemical (IHC) analyses of orthotopic tumors showed significantly reduced TGF-beta signaling, increased CD31 and VEGFA staining in tumors of FETα/DNRII cells as compared to those of vector control cells. These results indicate that inhibition of TGF-beta signaling increases VEGFA expression and angiogenesis, which could potentially contribute to enhanced metastasis of those cells *in vivo*. IHC studies performed on human colon adenocarcinoma specimens showed that TGF-beta signaling is inversely correlated with VEGFA expression, indicating that TGF-beta-mediated suppression of VEGFA expression exists in colon cancer patients.

## Introduction

Transforming growth factor beta (TGF-beta) comprises a group of multifunctional polypeptides that regulate different cellular processes, including cell proliferation, apoptosis, differentiation, migration, tumorigenecity and metastasis, through binding to TGF-beta receptors. Many studies indicate that TGF-beta signaling acts as either a tumor promoter or a tumor suppressor. The tumor promoter function of TGF-beta has been associated with its ability to induce an epithelial to mesenchymal transition (EMT), which confers resistance to the apoptotic effects of TGF-beta [1]–[3]. However, we and others have demonstrated experimentally that TGF-beta mediates tumor suppressor activity in a variety of cancers including colon cancer, and that loss of TGF-beta signaling leads to acquisition and progression of malignancy [4]–[11]. Our recent studies demonstrate that TGF-beta signaling suppresses metastasis in a subset of colon cancer cells in an orthotopic model *in vivo* [12]. Therefore identifying the mechanism(s) by which TGF-beta elicits its metastasis suppressor function is crucial for our understanding of tumor progression and for developing effective therapeutic strategies.

Angiogenesis is an important determinant of tumor progression. Solid tumors cannot grow beyond a certain size without sufficient vascular supply to provide enough oxygen and nutrients. The VEGF/VEGFR pathway is a key mediator of angiogenesis [13] and VEGFA acts as a potent tumor angiogenic factor [14]. VEGFA stimulates the growth of new blood vessels, which provide tumors with needed oxygen and nutrients [14], [15]. Expression of VEGFA has been shown to be regulated at the transcriptional and translational levels [16]–[19]. Regulation of VEGFA expression by TGF-beta signaling has not been very well studied. It has been reported that TGF-beta induces VEGF secretion in human cytotrophoblast cells [20]. On the contrary, another group showed that TGF-beta antisense oligonucleotides increases VEGFA expression in human keratinocytes and skin fibroblasts *in vitro* [21]. Therefore, TGF-beta may differentially regulate VEGFA expression dependent upon cellular context.

The FET colon carcinoma cell line, which was isolated from a well differentiated early stage cancer, retains autocrine TGF-beta activity. These cells form a small tumor nodule at the site of inoculation in athymic nude mice that does not progressively grow and then regresses over 3–5 weeks [5], [22]. The inability of these colon carcinoma derived cells to generate progressive tumor growth *in vivo* suggests that, relative to the more progressed model cell lines, FET cells retain many normal growth controls, including the inhibitory response to TGF-beta. Therefore, FET cells were engineered to express TGF-α (FETα), which constitutively activates EGFR/ErbB signaling. These cells (FETα) readily formed xenograft tumors in nude mice [22]. However, FETα cells retained their response to TGF-beta-mediated growth inhibition and rarely metastasized *in vivo*. Expression of a dominant negative TGF-beta type II receptor (DNRII) in FETα cells, designated FETα/DNRII, abrogated responsiveness to TGF-beta-induced growth inhibition and apoptosis under stress conditions *in vitro* and significantly increased metastatic potential in an orthotopic model *in vivo* [12]. Thus, it appears that the loss of TGF-beta tumor suppressor response is permissive for the development of metastasis by FETα cells, which provides evidence in support of the metastasis suppressor function of TGF-beta signaling in this cell model. The current study utilizes the FETα/vector and FETα/DNRII cell model system to test the hypothesis that TGF-beta signaling mediates angiogenesis through regulating VEGFA expression, which could contribute to the metastasis suppressor function of TGF-beta. In this study, we report that, in colon cancer cells, TGF-beta inhibits VEGFA expression at the post-transcriptional level, probably by decreasing VEGFA protein stability through ubiquitination and degradation. This regulation is Smad3-dependent and Smad2-independent. Our data also indicate that TGF-beta-mediated down-regulation of VEGFA expression is through a PKA-mediated pathway. In addition, we demonstrate that the loss of autocrine TGF-beta in FETα/DNRII cells results in increased expression of VEGFA as compared to the control cells both *in vitro* and *in viv*o, suggesting that one aspect of autocrine TGF-beta activity in its metastasis suppressor context represses angiogenesis. Finally, IHC studies of sections of human colonic adenocarcinomas reveal that TGF-beta signaling is inversely correlated with VEGFA expression, indicating that the link between loss of TGF-beta activity and increased VEGFA expression also exists in colon cancer patients.

## Materials and Methods

All experiments involving animals were approved by the University of Nebraska Medical Center Institutional Animal care and Use Committee (IACUC #: 07-043-08-FC).

### Cell Lines and Reagents

The FETα/DNRII cells were obtained by transfection of a dominant negative TGF-beta type II receptor into FETα cells, whereas FETα/vector control cells were generated by transfection of a control plasmid. FETα/vector, FETα/DNRII, HCT116, RKO, C, CBS, GEO and parental FET colon carcinoma cells [9] were cultured at 37°C in a humidified incubator with 5% CO_2_ in McCoy's 5A serum-free medium (Sigma) supplemented with 5 ng/ml epidermal growth factor, 20 µg/ml insulin, and 4 µg/ml transferrin as described previously [23]. Recombinant human TGF-beta1 was purchased from R&D Systems. Cycloheximide, MG132 and PYR41 were purchased from AMRESCO, Sigma and CALBIOCHEM respectively.

### Western Blot Analysis

Cells were lysed in NP40 lysis buffer (50 mM Tris-HCl, pH 7.4, 150 mM NaCl, 0.5% NP-40, 50 mM NaF, 1 mM NaVO_3_, 1 mM phenylmethylsulfonyl fluoride, 1 mM dithiothreitol, 25 µg/ml aprotinin, 25 µg/ml trypsin inhibitor and 25 µg/ml leupeptin) at 4°C and used for western blot analyses as previously described [24], [25]. In brief, total protein from cell lysates (30 to 60 µg) was separated by SDS-PAGE and transferred to a nitrocellulose membrane. The proteins were detected using an enhanced chemiluminescence system (Amersham Biosciences). Antibodies for western blots analyses of VEGFA, Smad2/3, pSmad2/3, PKA and actin were obtained from Santa Cruz Biotechnology Inc, Abcam, Chemicon, Cell Signaling Technology and Sigma respectively.

### Isolation of RNA and RT-PCR

Total cellular RNA from FET cells were isolated using Trizol total RNA isolation reagent (Invitrogen), according to the manufacturer's instructions. For RT-PCR, 2 µg of RNA was reverse-transcribed with M-MLV reverse transcriptase (Promega,) using a random primer. Two microliters of the cDNA product was used to amplify human VEGFA. Primer sequences for VEGFA were 5′-CGGATCAAACCTCACCAAGGCC-3′ (forward) and 5′-CTTTCTCCGCTCTGAGCAAGGC-3′ (reverse). Conditions for amplification were: one cycle at 94°C for 2 minutes followed by 30 cycles at 94°C for 30 seconds, 60°C for 30 seconds and 72°C for 30 seconds. The actin gene was used as an internal control.

### Immunofluorescence Staining

The FETα/vector and FETα/DNRII cells were grown to 70–80% confluent on glass cover slips. They were washed with PBS and fixed in ice cold methanol for 5 minutes. Fixed cells were permeabilized with 0.1% Tween 20 in PBS and blocked for nonspecific binding with 10% normal goat serum in PBS for 30 minutes at 4°C. Cells were then incubated with primary anti-VEGFA antibody (1∶50 dilution, Santa Cruz Biotechnology) for overnight at 4°C, followed by incubation in the dark for1 hour at room temperature with the secondary antibody Dylight488-conjugated with anti-rabbit IgG (1∶400 dilution, Jackson ImmunoResearch Laboratories). Stained cells were mounted in vectashield hardset mounting medium with dapi (Vector Labs) and imaged using a fluorescent microscope.

### Tissue Samples

Formalin fixed paraffin embedded (FFPE) tissue blocks from the primary tumors of mice bearing FETα/vector and FETα/DNRII cells were described previously [2]. FFPE blocks of normal human colon removed for reasons other than malignancy and those containing invasive colonic adenocarcinomas were obtained from files of the Department of Pathology and Microbiology at the University of Nebraska Medical Center (UNMC). The ages of all patients were between 55 and 85 years, with material coming from both men and women. The cancer patients did not receive anticancer therapy prior to surgical removal of the tumor.

The study was performed with the approval of the ethics committee (Institutional Review Board) of UNMC. Tissue used for the study was obtained from excess material remaining in the paraffin block after issuance of the pathologic diagnosis. The surgery consent form used at UNMC includes a provision allowing the use of excess material for research purposes. Each form is on file with the Department of Pathology and Microbiology and was reviewed prior to experimentation. Therefore, the ethics committee waived the need for additional written consent from the participants.

### Immunohistochemistry (IHC) Staining

Four micron thick tissue sections were cut and dewaxed in histoclear for 15 minutes and then rehydrated using 100%, 95% and 70% of graded alcohol for 5 minutes respectively. Antigen retrieval for pSmad2 was performed using Novocastra, Epitope Retrieval Solutions at pH 6.0 (Leica). Incubation with primary anti-pSmad2 antibody (1∶200 dilution, Millipore) followed by NovoLink Polymer (Leica) was used to detect phosphorylation of Smad2. Antigen retrieval for VEGFA and CD31 was performed using Tris-EDTA buffer at pH 9.0. To detect VEGFA and CD31 expression, anti-VEGFA antibody (1∶50 dilution, Santa Cruz) and anti-CD31 antibody (1∶100 dilution, Abcam) were incubated for overnight at 4°C, followed by incubation with a HRP conjugated goat anti-rabbit secondary antibody (1∶300 dilution, Jackson Laboratories) for VEGFA and with a HRP labeled polymer anti-rabbit (DAKO) for CD31. To neutralize endogenous peroxidase, Dako Dual Endogenous Enzyme Block (DAKO) was used for VEGFA and CD31 while Novocastra Peroxidase Block (Leica) was used for pSmad2. Slides were then developed using DAB Chromogen kit (DAKO) followed by counter-staining with hematoxylin. Slides were mounted with Permount (Fisher Scientific) and subjected to microscopic examinations. Staining density was measured and quantified with ImagePro plus 7.0 Software using 20× or 40× images.

## Results

### TGF-beta signaling inhibits VEGFA expression in colon cancer cells

**Figure 1:**
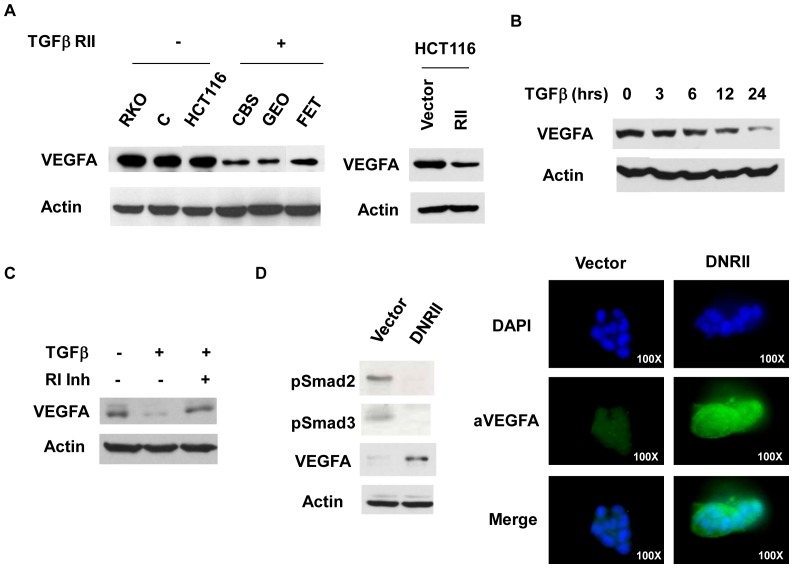
TGF-beta inhibits VEGFA expression in colon cancer cell lines. (A) Western blot analysis of VEGFA expression in different colon cancer cell lines and HCT116 cells re-expressing TGF-beta RII. (B and C) FET cells were grown to 50% confluent and treated with 4 ng/ml TGF-beta1 for the indicated time periods (B) or with 4 ng/ml TGF-beta1 in the presence or absence of 200 nM SB525334 for 48 hrs (C). Western blot analyses were performed with an anti-VEGFA antibody to detect VEGFA expression. β-actin was used as a loading control. (D) Western blot (left panel) and immuno-staining (right panel) analyses of VEGFA expression in FETα/vector and FETα/DNRII cells. Phosphorylation of Smad2 and Smad3 (pSmad2 & pSmad3) was shown to indicate Smad activation (left panel).

Examination of VEGFA expression in a panel of colon cancer cells indicated that cell lines with the wild type TGF-beta RII (CBS, GEO and FET [7]) expressed lower levels of VEGFA than those with mutant TGF-beta RII (RKO, C, HCT116 [7]) (Fig. 1A, left panel), suggesting an inverse relationship between TGF-beta signaling and VEGFA expression. Re-expression of the wild type TGF-beta RII in HCT116, one of the cell lines with mutant TGF-beta RII, led to reduced VEGFA expression (Fig. 1A, right panel). To directly determine whether TGF-beta signaling negatively regulates VEGFA expression, FET cells were treated with 4 ng/ml of TGF-beta. As shown in Figure 1B, exogenous TGF-beta reduced VEGFA expression in a time-dependent manner, with significant reduction after 24 hours of TGF-beta treatment. SB525334, a potent inhibitor of TGF-beta type I receptor (RI) kinase, was used to confirm the effect of TGF-beta. Treatment of FET cells with SB525334 reversed the inhibitory effect of TGF-beta on expression of VEGFA (Fig. 1C). To further confirm the suppressive effect of TGF-beta on VEGFA expression, a dominant negative TGF-beta RII (DNRII) was introduced into FETα cells to abrogate endogenous TGF-beta signaling, which was shown by reduced Smad2 and Smad3 phosphorylation (pSmad2 & pSmad3; Fig. 1D, left panel). Consequently, VEGFA expression was increased significantly in DNRII cells as compared to the vector control cells (Fig. 1D, left panel). Immunofluorescence staining of VEGFA confirmed higer expression of VEGFA in FETα/DNRII cells than in FETα/vector cells (Fig. 1D, right panel).

### TGF-beta inhibits VEGFA expression at the post-transcriptional level

**Figure 2:**
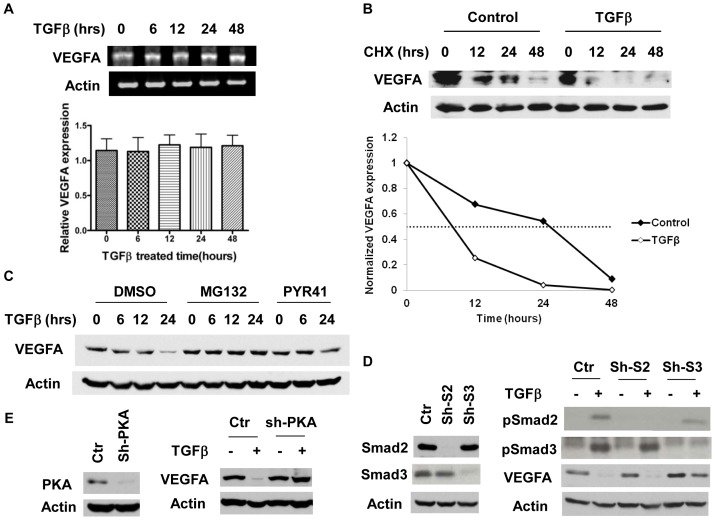
TGF-beta regulates VEGFA expression at the post-transcriptional level. (A) FET cells were grown to 50% confluent and treated with 4 ng/ml TGF-beta1 for the indicated time periods. RT-PCR was performed as described in *Materials and Methods* (upper panel). The intensity of each VEGFA mRNA band was quantified and normalized with that of the corresponding actin band. Values are means ± S.E. from triplicate experiments (lower panel). (B) Western blot analysis of VEGFA expression was performed at the indicated time points in FET cells treated with 100 µg/ml cycloheximide (CHX) alone or CHX and TGF-beta together (upper panel). The intensity of each VEGFA band was quantified and normalized with that of the corresponding actin band (lower panel). (C) Western blot analysis of VEGFA expression in FET cells treated with TGF-beta alone, TGF-beta with MG132 (10 µM) or TGF-beta with PYR41 (15 µM). (D) Expression of Smad2 and Smad3 in FET cells with empty vector control (Ctr), Smad2 shRNA (Sh-S2) or Smad3 shRNA (Sh-S3) was shown in the left panel. Phosphorylation of Smad2 and Smad3 and expression of VEGFA in above cells treated with 4 ng/ml of TGF-beta was shown in the right panel. (E) Expression of PKACatα in FET cells with empty vector control (Ctr) or PKACatα shRNA (Sh-PKA) was shown in the left panel. VEGFA expression in above cells treated with 4 ng/ml of TGF-beta for 48 hours was shown in the right panel.

To determine how TGF-beta suppresses VEGFA expression, mRNA expression of VEGFA was determined in FET cells treated with TGF-beta for different periods of time. RT-PCR results showed that VEGFA mRNA levels remained unchanged after TGF-beta treatment for up to 48 hours (Fig. 2A, upper panel). Quantification of repeated experiments confirmed the observation (Fig. 2A, lower panel). These results indicate that TGF-beta regulates VEGFA expression at the post-transcriptional level. One of the possible mechanisms of post-transcriptional regulation is that of protein stability. To determine whether TGF-beta signaling regulates the protein stability of VEGFA, cycloheximide (CHX) was added to FET cells to inhibit protein synthesis while they were treated with TGF-beta. Under these conditions, levels of VEGFA decreased faster in TGF-beta-treated samples than in control samples (Fig. 2B, upper panel). Quantification and normalization of the intensity of the VEGFA bands with that of the corresponding actin bands clearly showed that TGF-beta treatment reduced the half-life of VEGFA protein (Fig. 2B, lower panel), indicating that TGF-beta regulates VEGFA protein stability. To further dissect the underlying mechanism, a proteasome inhibitor, MG132, or ubiquitin E1 inhibitor, PYR41, was used to treat FET cells together with TGF-beta. As shown in Figure 2C, either MG132 or PYR41 prevented TGF-beta-mediated suppression of VEGFA expression. These results suggest that TGF-beta signaling decreases VEGFA protein stability through ubiquitination and degradation.

TGF-beta signaling is mediated predominantly through Smad activation. However, Smad-independent TGF-beta signaling has also been reported in different cell types [23], [24]. To determine whether TGF-beta-mediated inhibition of VEGFA expression is Smad-dependent or -independent, Smad2 and Smad3 were knocked down individually in FET cells by shRNAs specific for Smad2 or Smad3. Expression of Smad2 or Smad3 was reduced efficiently and specifically in Smad2 or Smad3 knockdown cells (Fig. 2D, left panel). As a result, increases in pSmad2 or pSmad3 upon TGF-beta treatment were abolished in Smad2 or Smad3 knockdown cells respectively (Fig. 2D, right panel). Western blot analysis indicated that inhibition of VEGFA protein expression by TGF-beta was partially reversed in Smad3 knockdown cells whereas knockdown of Smad2 had little effect (Fig. 2D, right panel). These results indicate that TGF-beta suppresses expression of VEGFA in a Smad3-dependent and Smad2-independent manner. The partial reversal of TGF-beta-mediated suppression of VEGFA expression in Smad3 knockdown cells may be attributed to incomplete silencing of Smad3 expression in those cells or the existence of a Smad3-independent mechanism.

As shown previously, TGF-beta regulates XIAP downregulation through a PKA-mediated pathway [25]. We next determined whether PKA is involved in the regulation of VEGFA expression by TGF-beta. The expression of PKA catalytic α subunit (PKACatα) was knocked down by a specific shRNA in FET cells (Fig. 2E, left panel). As a result, TGF-beta-mediated inhibition of VEGFA expression was almost completely abrogated in those cells (Fig. 2E, right panel). Taken together with the observation that PKA activation is Smad3-dependent [25], these results indicate that TGF-beta/Smad3/PKA signaling regulates downregulation of VEGFA expression in colon cancer cells.

### TGF-beta signaling inhibits VEGFA expression and suppresses tumor angiogenesis in vivo

**Figure 3:**
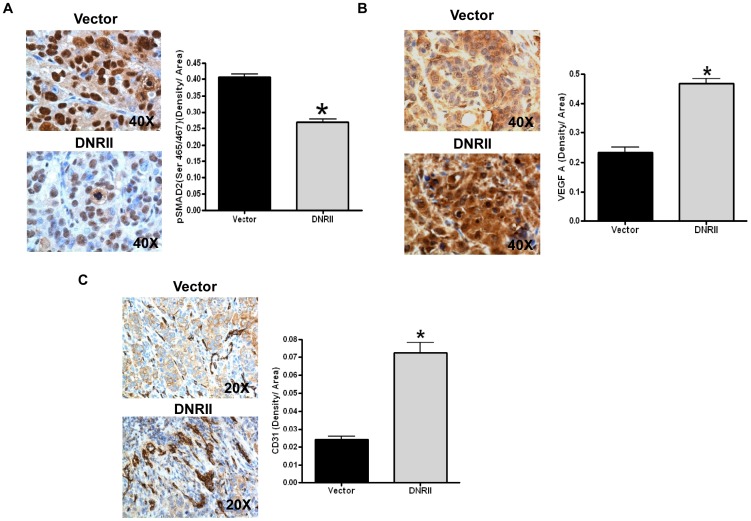
TGF-beta signaling inhibits VEGFA expression and suppresses tumor angiogenesis *in vivo*. Representative images of IHC staining of phospho-Smad2 (A), VEGFA (B) and CD31 (C) in the primary tumors of FETα/vector and FETα/DNRII cells (left panels). Staining density was measured and quantified with ImagePro plus 7.0 Software using 20× or 40× images (right panels). Four animals were analyzed for each type of cells. Fifteen to twenty histologically similar fields were randomly selected from each slide for analysis of staining density. The data are presented as the mean ± SE. *P* values were calculated using Student's *t*-test. * *P*<0.001.

VEGFA is a key regulator of blood vessel formation and plays an important role in angiogenesis, which contributes to tumor development and progression. We have shown above that TGF-beta signaling inhibits VEGFA expression (Fig. 1B). Since TGF-beta acts as a metastasis suppressor in colon cancer cells [12], we examined whether suppression of VEGFA expression by TGF-beta contributes to metastasis suppression function of TGF-beta. We used FETα/vector and FETα/DNRII cell model described previously [12] to address this question. VEGFA expression was much higher in FETα/DNRII cells than that in FETα/vector cells *in vitro* due to abrogation of TGF-beta signaling (Fig. 1D). As shown previously, FETα/DNRII cells showed significantly increased metastatic potential as compared to FETα/vector cells in an orthotopic model *in vivo* [12]. Primary tumor sections prepared from each mouse orthotopically transplanted with FETα/vector or FETα/DNRII cells were examined for TGF-beta signaling. Since FETα cells express both Smad2 and Smad3 (data not shown), Smad2 phosphorylation was used as an indicator for active TGF-beta/Smad2/3 signaling. IHC analyses using an anti-phospho-Smad2 (anti-pSmad2) antibody showed that nuclear Smad2 phosphorylation was significantly reduced in the primary tumors of FETα/DNRII cells as compared to those of FETα/vector cells (Fig. 3A, left panel). Quantification of staining density of nuclear pSmad2 in multiple samples indicated that TGF-beta signaling was inhibited in the tumors of FETα/DNRII cells by about 40% (Fig. 3A, right panel, * *P*<0.001). To determine whether TGF-beta signaling suppresses VEGFA expression *in vivo*, we examined VEGFA expression in the primary tumors using IHC analyses. Results showed that VEGFA expression was much higher in the tumors of FETα/DNRII cells than those of the control cells (Fig. 3B, left panel). Quantification of staining density of VEGFA in multiple samples indicated that VEGFA expression was increased by more than two-fold in these tumors (Fig. 3B, right panel, * *P*<0.001), confirming the suppressive role of TGF-beta in VEGFA expression *in vivo*. To determine whether VEGFA expression has any biological consequences *in vivo*, we next analyzed microvessel formation within the tumors using an anti-CD31 antibody. CD31 is a marker of blood vessel endothelial cells. In the tumors of FETα/DNRII cells, microvessel density was more than 3-fold higher than that observed in the control samples (Fig. 3C, * *P*<0.001). These results indicate that inhibition of TGF-beta signaling leads to elevated VEGFA expression, which contributes to increased angiogenesis. Taken together with the results of increased metastatic potential of FETα/DNRII cells in the orthotopic model, our studies suggest that TGF-beta signaling suppresses VEGFA-mediated angiogenesis and tumor progression.

### Expression of VEGFA inversely correlates with TGF-beta signaling in human colon cancer adenocarcinoma

**Figure 4:**
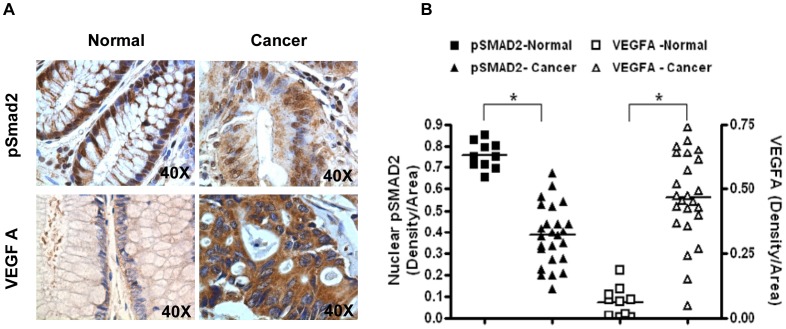
Expression of VEGFA inversely correlates with TGF-beta signaling in human colon cancer specimens. Sections prepared from 10 normal colon and 24 individual colon cancer patient specimens were stained using anti-phospho-Smad2 and anti-VEGFA antibodies. (A) Representative images of IHC staining of phospho-Smad2 and VEGFA in normal colon and colon cancer specimens. (B) Staining density was measured and quantified with ImagePro plus 7.0 Software using 40× images in each sample. Normal colon samples are represented by squares and colon cancer samples by triangles. Bars represent mean values of each group of samples. *P* values were calculated using Student's *t*-test. * *P*<0.001.

The above *in vitro* and *in vivo* results demonstrate that TGF-beta negatively regulates VEGFA expression in human colon cancer cell lines. We next examined whether there was any correlation between TGF-beta signaling and VEGFA expression in human colonic adenocarcinomas. Sections prepared from 10 normal colon and 24 individual colon cancer patients were examined for active TGF-beta signaling and VEGFA expression. Since Smad2 and Smad3 are rarely mutated in colon cancer [26], Smad2 phosphorylation was used in this study to indicate active TGF-beta/Smad2/3 signaling. Normal human colon showed strong and distinct nuclear staining for Smad2 phosphorylation and weak staining for VEGFA in the crypt epithelial cells whereas the malignant epithelial cells in the adenocarcinoma cases displayed at most weak and diffused staining for Smad2 phosphorylation and strong staining for VEGFA by IHC analyses (Fig. 4A). Figure 4B shows the quantification of staining density of nuclear Smad2 phosphorylation and VEGFA expression of individual patient samples. Overall, normal colonic epithelial cells display higher nuclear Smad2 phosphorylation and lower VEGFA expression than adenocarcinomas, supporting the view that expression of VEGFA inversely correlates with TGF-beta signaling in human colonic adenocarcinoma.

## Discussion

TGF-beta signaling has been shown to suppress tumor formation and metastasis in a subset of colon cancer cells through many different mechanisms including inhibition of cell proliferation and induction of apoptosis [4], [12], [25], [27]. The ability of malignant cells to withstand environmental stresses, particularly those associated with metastasis, is considered a key factor in tumor development and progression [28]. Since environmental restriction on growth is very common in solid tumors such as colon carcinoma, a mechanism that enables increased angiogenesis to provide sufficient nutrients and oxygen would be highly advantageous to malignant cells and enhance their aberrant survival capacity. VEGFA is a potent angiogenic factor that plays essential roles in cancer. Tumor cells secret VEGFA to induce vasculature to develop, allowing a sufficient supply of oxygen and nutrients [14]. Therefore, VEGFA could be a potential target for cancer therapy. We have shown previously that escape from TGF-beta-mediated apoptosis in colon cancer cells contributes to their increased survival capacity and enhanced metastatic potential *in vivo* [12]. We show here that, in addition to inducing apoptosis, TGF-beta signaling can also inhibit expression of VEGFA and as a consequence, angiogenesis in colon cancer cells *in vivo* and that abrogation of TGF-beta enables increased VEGFA expression and angiogenesis, which could facilitate tumor growth and metastasis development. Furthermore, an inverse relationship between TGF-beta signaling and VEGFA expression is also observed in human colon cancer specimens, indicating the relevance of our studies to human cancer. Therefore, the inhibitory effect of TGF-beta on VEGFA expression and angiogenesis provides another important and novel mechanistic basis for TGF-beta tumor and metastasis suppressor function.

Although expression of VEGFA can be regulated at the transcriptional level by HIF1α [19], its regulation by TGF-beta is at the post-transcriptional level (Fig. 2A). Our results indicate that TGF-beta treatment reduced the stability of the VEGFA protein through ubiquitination and degradation (Fig. 2B & 2C). Moreover, TGF-beta-mediated decrease in VEGFA expression occurs via a PKA- and Smad3-dependent and Smad2-independent pathway (Fig. 2D & 2E). It has been shown that activation of PKA by TGF-beta involves Smad3 but not Smad2 and that PKA activation promotes protein degradation through ubiquitination [25]. Therefore, it is possible that TGF-beta mediates VEGFA degradation through the Smad3/PKA pathway. This is a novel mechanism by which expression of VEGFA is regulated by TGF-beta signaling. Further studies will be necessary to determine the underlying mechanism.

High frequency of Smad4 mutation and inactivation is closely associated with increased metastases and poor prognosis in colon cancer [29], [30]. Although TGF-beta signaling acts as a tumor suppressor in a subset of colon cancer cells (i.e. FET, GEO and CBS, etc) that express wild type Smad4 [4], [10], [11], Smad4-independent TGF-beta signaling has been shown to promote colon cancer metastasis [31]. It has been reported that TGF-beta induces VEGF expression in Smad4-null colon cancer cells [32], suggesting that TGF-beta-mediated activation of Smad4-independent pathways (i.e. MEK-Erk and p38-MAPK, etc) is involved in the up-regulation of VEGF expression, which may cooperate with other pro-oncogenic pathways to promote metastasis. Therefore, the opposite effect of TGF-beta on VEGFA expression could contribute to its differential role as tumor suppressor or tumor promoter in different cell context.

In summary, we have identified a novel mechanism by which TGF-beta suppresses VEGFA expression, angiogenesis and metastasis in a subset of colon cancer cells. This study is significant since it has been shown that expression of VEGFA is increased in colon cancer and is associated with disease localization, stage and long-term disease-specific survival [33]. Avastin, a monoclonal antibody directed against VEGFA, has been widely used with chemotherapy to treat metastatic colon cancer with improved efficacy [34], [35]. However, some patients eventually develop resistance to the treatment [36]. Therefore, understanding how VEGFA expression is regulated will facilitate development of novel strategies to effectively target it for cancer treatment. Our studies suggest that activation or restoration of TGF-beta signaling in a sub-population of colon cancer patients may improve their response to anti-VEGFA therapies.
